# A 13 cm asymptomatic abdominal aortic aneurysm complicated with arterial obstruction in the left lower limb: A Case report

**DOI:** 10.1016/j.amsu.2021.102815

**Published:** 2021-09-04

**Authors:** Sarya Swed, Salim Tfankji, Hussein Alkanj, Tasneem Mohamed, Nawras Alhalabi, Hidar Alibrahim

**Affiliations:** a Faculty of Medicine, Aleppo University, Aleppo, Syria; b Department of Vascular Surgery, Aleppo University Hospital, Aleppo University, Aleppo, Syria; c General Physican in The National Ribat University Hospital, Sudan; d Faculty of Medicine, Damascus University, Damascus, Syria; e Faculty of Medicine, Syrian Private University, Damascus, Syria

**Keywords:** Abdominal aortic aneurysm, Arterial obstruction, Case report

## Abstract

Abdominal aortic aneurysm is often asymptomatic when it is small to medium in size, up to a maximum of 8 cm, in this case, a patient presented to the vascular surgery department with a complaint of acute pain in the left lower extremity with pallor due to the presence of arterial obstruction. On physical examination, we found a large pulsating mass in the abdomen. After performing a multislice computed tomography, we confirmed the presence of aneurysm with diameters of 13 × 8 cm below the level of the renal arteries. The rarity of this case in the medical literature come from that the aneurysm has reached this size and did not even rupture and did not cause any digestive discomfort or abdominal pain only complicated with an arterial obstruction in the lower left extremity as a result of the dissection that occurred. An emergency surgical operation was performed to remove the aneurysm and install the Dacron joint, and the patient was placed for 24 hours under intensive care and discharged after 5 days to have excellent results with the patient's condition improving without any complications after the operation. In conclusion, aneurysms constitute a serious condition facing vascular surgeons, especially if they are large in size without any symptoms.

## Highlights

• Aortic aneurysms are asymptomatic in most cases.
• When the aneurysm expands, symptoms such as abdominal pain and back pain may appear.
• The extreme expansion led to dissociation and arterial obstruction of the left lower limb without typical symptoms.

## 1 Introduction

The aneurysm is an abnormal local dilatation in the wall of blood vessel, usually an artery, that develops as a consequence of a defect, disease or injury. The most common site for aneurysm development is in the infrarenal aorta, called abdominal aortic aneurysms (AAA) [1]. The most important risk factors are smoking, male gender and family history, whereas interestingly, diabetes mellitus is a negative risk factor for AAA [2]. Most patients with AAA are asymptomatic this is commonly an undiagnosed condition however, Symptomatic aneurysms typically present with pain localised to the abdomen, back and flank and digestive discomfort [3]. A very small fraction of patients with intact asymptomatic AAA are diagnosed with a pulsatile mass at a clinical examination or because the large AAA compresses other intraabdominal organs. Computed tomography (CT) scan will support the diagnosis, detect possible concurrent aneurysmal disease in other vessels, and give the possibility to plan the surgical intervention, weather open or endovascular aneurysm repair (EVAR); moreover, magnetic resonance imaging (MRI) cannot be performed in emergency situations. AAA enlarge gradually and as the size increases, so does the risk of rupture. Rupture occurs when the mechanical stress acting on the wall exceeds the wall strength, with rupture of the aneurysm causing intraabdominal hemorrhage. Patients with an asymptomatic AAA of >5.5 cm in diameter should be considered for elective repair, while surveillance is recommended for smaller AAA. Treatment is through open surgery to exclude the AAA from the systemic circulation and installing a stent-graft from the healthy end of the artery to the other healthy end.

Herein, we present and discuss a rare case of a 13 cm asymptomatic abdominal aortic aneurysm complicated with acute arterial obstruction in the left lower limb.

This case report has been reported in line with the SCARE criteria 2020 [6].

## 2 Case presentation

A 54-year-old male presented to surgical emergency room of Aleppo University Hospital with the complaints of sharp pain in the left lower extremity with pallor and coldness, in addition to the presence of coldness with no pulse in the lower right extremity, these symptoms are consistent with the presence of acute arterial obstruction in the left lower extremity.

During the clinical examination of the abdomen, a large and pulsating mass was noted from the abdomen wall. Despite the presence of this very large pulsating mass, the patient did not complain of any digestive symptoms such as digestive discomfort, abdominal pain or bloody defecation. The clinical history indicated the existence of a previous surgical operation to repair anal hemorrhoids 8 years ago, in addition to the presence of orally treated systemic hypertension. Notably, the patient smoked an average of one pack per day for 40 years.

Initial blood tests included a white blood cell count of 8.8 × 10^9/L, hemoglobin level 10.7, platelets count 171 × 10^3/UL. Laboratory tests for electrolytes, liver and kidney enzymes were within the normal ranges.

**Fig. 1:**
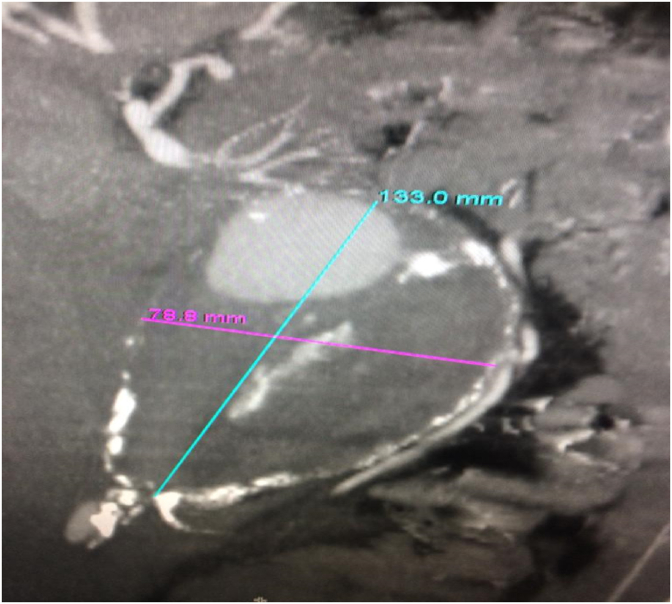
Multislice CT scan showing the dimensions of the aneurysm; 13 × 8 cm.

**Fig. 2:**
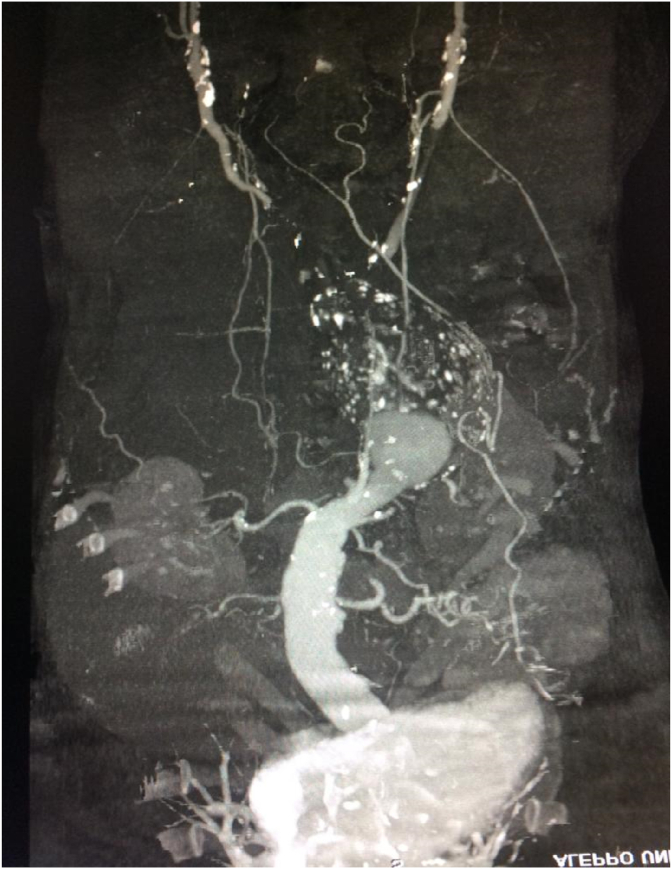
Multislice CT scan showing the dimensions of the aneurysm.

**Fig. 3:**
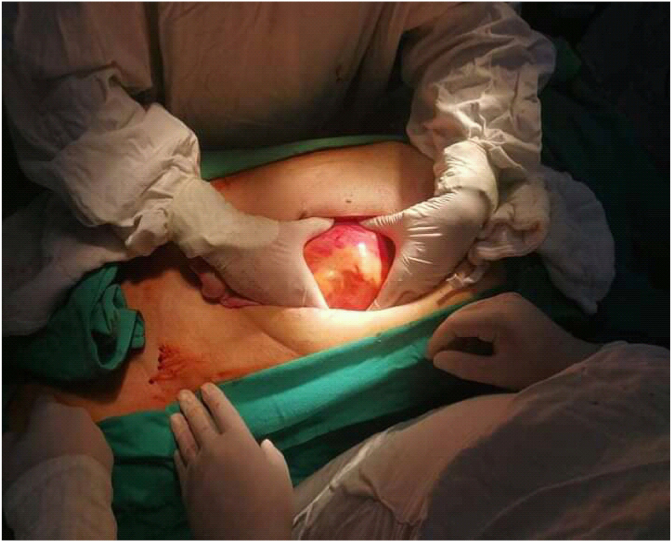
Excision of the dilated aneurysm during the operation.

**Fig. 4:**
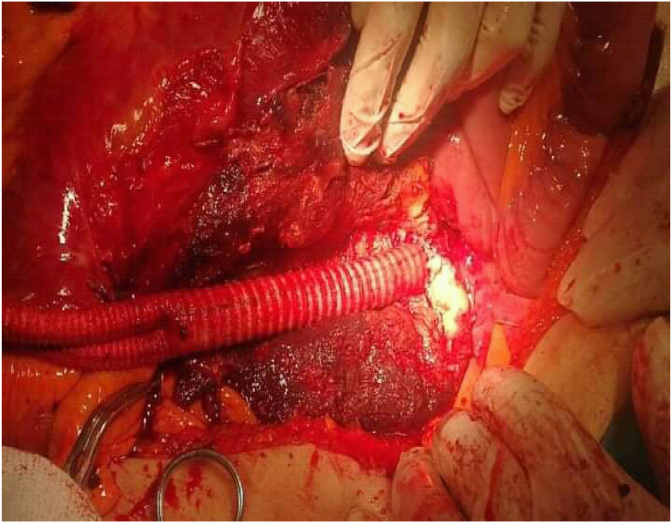
Double dacron aortofemoral joint insertion.

A multislice CT scan with contrast was performed showing the presence of a dissected aneurysm of approximately 8 × 13 cm with complete obstruction of the abdominal aortic lumen below the renal arteries (Fig. 1, Fig. 2). The open surgery was performed with the participation of four doctors from the vascular surgery department, an anesthesiologist and a nurse. We used halothane for general anesthesia. We removed a large aneurysm (Fig. 3), and installed a 16 × 8 double aortofemoral dacron joint (Fig. 4). The operation took 2 h, We did not face any difficulties during the surgery, but we were very careful to deal sensitively with aneurysms of this large size. A surgical drain was installed. Fortunately, it was within 24 hours, as no purulent or serous oozing was noticed. To avoid infection of the wounds after surgery, metronidazole was given with ceftriaxone intravenously, with intravenous paracetamol for pain relief. The patient was admitted to the intesive care unit for the first day, then he was transferred to the surgery department. He was discharged after 5 days. The patient's condition was followed up for 3 months by telemedicine, no complications were observed and the results were very acceptable.

## 3 Discussion

AAA is functionally defined by progressive changes in the arterial wall in response to changes in arterial pressure, leading to thinning of the wall commonly below the level of the renal arteries [4]. The aneurysms are often asymptomatic if they are of acceptable size, that is, it is very rare for the aneurysm to reach a diameter of 13 cm without giving any typical symptoms such as abdominal pain or any symptom related to the digestive tract, and this is what we saw in our case. The patient did not feel any prior symptoms until after the complication of the aneurysm with an arterial obstruction as a result of the dissection and the growing size of AAA, the greater the possibility of rupture, which is an acute emergency condition. The mortality for patients with ruptured AAA is 65–85% and about half die before being admitted to hospital. Diagnosis is inevitably confirmed by multislice CT scan for asymptomatic aneurysms, and sometimes with clinical examination. AAA with a diameter of more than 4.5 cm in women and 5.5 cm in men should be treated surgically quickly to avoid rupture and cardiogenic shock from hemorrhage and hypotension. The treatment is done by surgical intervention on the aneurysm to remove it and install a Dacron or Polytetrafluoroethylene-covered stent grafts and surveillance protocols are commonly used at most vascular departments for patients with asymptomatic AAA and also indicate when patients should be evaluated for aortic repair [5].

## 4 Conclusion

Aneurysms should be carefully evaluated, especially asymptomatic, and considered as a serious, silent condition if they appear in a large size, as they can rupture at any time and cause hypovolemic shock. In our case, the aneurysm occupied a diameter of 13 cm without any symptoms, and it was not discovered until after it was complicated with an arterial obstruction.

## Conflicts of interest

All authors declared no conflict of interest.

## Sources of funding

This research did not receive any specific grant from funding agencies in the public, commercial, or not-for-profit sectors.

## Ethical approval

This case reports didn't require review by Ethics committee, it was approved for publication by the Faculty of Medicine, Aleppo University, Aleppo Syria, and The National Ribat University Hospital, Ribat, Sudan.

## Registration of research studies

Not applicable.

## Consent of Patient

A written informed consent was obtained from the patient for publication of this case reports and accompanying images. A copy of the written consent is available for review by the Editor-in-Chief of this journal.

## Sources of funding

This research did not receive any specific grant from funding agencies in the public, commercial, or not-for-profit sectors.

Sarya Swed: contributed in study concept and design, data collection, and writing the paper.

Salim tfankji: contributed in data interpretation and writing the paper.

Hussien alkanj:contributed in data interpretation and writing the paper.

Tasneem Mohamed: contributed in writing the paper.

Nawras Alhalabir:contributed in writing the paper.

Haidar Alibrahim:contributed in writing the paper.

Yamane Chawa: contributed in writing the paper.

## Registration of research studies

Not applicable.

## Guarantor

Sarya Swed.

## Consent for Publication

All authored approved the publication of the final copy of this paper.
